# Estimating and Explaining the Differences in Health Care Seeking by Symptom Burden Among Persons With Presumptive Tuberculosis: Findings From a Population-Based Tuberculosis Prevalence Survey in a High-Burden Setting in India

**DOI:** 10.1093/ofid/ofae412

**Published:** 2024-07-19

**Authors:** Prathiksha Giridharan, Karikalan Nagarajan, Sriram Selvaraju, Asha Frederick, Esakkipriya Subbiah, Sasikumar Mani, Kannan Thiruvengadam, T S Selvavinayagam, Chandrasekaran Padmapriyadarsini, Tamil Nadu TB Prevalence Study Group, Havenesh Murugesan, Priya Rajendran, Makesh Kumar, Rajendran Krishnan, Paul Kumaran, J Chitra, V Rani, L Venkatesan, P Munivaradhan, D Nithyakumar, V Rameshbabu, P K Venkatramana, N Premkumar, S V Joseph Rajkumar, T Thangaraj, A Devanathan, P Balaji, T K Bharath, J Udayakumar, Wilkingson Mathew, John Arockia Doss, A Vasudevan, K Anbarasan, M Mahesh Kumar, P Kumaravel, P Chandrasekar, K Vasudevan, G Eswaran, R Krishna Bahadur, J Jeeva, E Duraivel, R Karunanidhi, S Kathiravan

**Affiliations:** ICMR–National Institute for Research in Tuberculosis, Chennai, India; Division of Infectious Disease Epidemiology, ICMR–National Institute of Epidemiology, Chennai, India; ICMR–National Institute for Research in Tuberculosis, Chennai, India; ICMR–National Institute for Research in Tuberculosis, Chennai, India; Directorate of Medical and Rural Health Services & State TB Cell, Chennai, India; ICMR–National Institute for Research in Tuberculosis, Chennai, India; ICMR–National Institute for Research in Tuberculosis, Chennai, India; ICMR–Regional Medical Research Centre, Port Blair, Andaman and Nicobar Islands; Directorate of Public Health and Preventive Medicine, Chennai, India; ICMR–National Institute for Research in Tuberculosis, Chennai, India

**Keywords:** decomposition, health seeking, India, symptoms, tuberculosis

## Abstract

**Background:**

There is a lack of research evidence on the quantitative relationship between symptom burden and health care seeking among individuals with presumptive tuberculosis (TB).

**Methods:**

Data were derived from a cross-sectional population-based TB survey conducted between February 2021 and July 2022 in 32 districts of India. Eligible and consented participants (age >15 years) underwent TB symptom screening and history elicitation. Fairlie decomposition analysis was used to estimate the net differences in health care seeking due to varied symptom burden—from 1+ burden (>1 symptom) to 4+ burden (>4 symptoms)—and decomposed by observable covariates based on logit models with 95% CIs.

**Results:**

Of the 130 932 individuals surveyed, 9540 (7.3%) reported at least 1 recent TB symptom, of whom 2678 (28.1%; 95% CI, 27.1%–28.9%) reportedly sought health care. The net differences in health care seeking among persons with symptom burden 1+ to 4+ ranged from 6.6 percentage points (95% CI, 4.8–8.4) to 7.7 (95% CI, 5.2–10.2) as compared with persons with less symptom burden. The presence of expectoration, fatigue, and loss of appetite largely explained health care seeking (range, 0.9–3.1 percentage points [42.89%–151.9%]). The presence of fever, cough, past TB care seeking, weight loss, and chest pain moderately explained (range, 5.3%–25.3%) health care seeking.

**Conclusions:**

Increased symptom burden and symptoms other than the commonly emphasized cough and fever largely explained health care seeking. Orienting TB awareness and risk communications toward symptom burden and illness perceptions could help address population gaps in health care seeking for TB.

## Teaser

- To explain poor health care seeking (<40%) for tuberculosis symptoms in India, we assessed the unknown relationship between symptom burden and health care seeking for tuberculosis.
- As the symptom burden increased, health care seeking for tuberculosis increased by 6.6 to 7.7 percentage points. The presence of cough and fever moderately explained health seeking while blood in sputum did not.
- Developing tailored risk communications to appraise symptom burden and elicit illness perceptions in the community could improve health care seeking for tuberculosis.

Delays in the diagnosis and treatment of tuberculosis (TB) lead to poor treatment outcomes for individuals who are infected and increase the transmission risk of TB [1]. In low- and middle-income countries, 42% of the patients had about a 1-month delay in seeking care for TB [2]. Early diagnosis and treatment of TB primarily depend on the health care–seeking behaviors of those who experience symptoms related to TB [2–4]. India notified almost 2.8 million TB cases in 2023, which accounted for one-fourth of global TB cases. Still, almost 18% of patients with TB are considered missing in India because they remain undiagnosed or not notified. In India, nearly half of all persons with TB symptoms seek care in the private sector, despite TB diagnosis and treatment being free of cost in public hospitals. Even among those who seek care for TB symptoms, the time taken for the initial diagnosis is almost 60 days in India.

Studies have documented that persons with TB symptoms have to navigate a complex pathway of public and private sector providers to arrive at a diagnosis, adding to further delays. Previous research has identified varied factors that influence the health care seeking for TB symptoms, which include poor awareness, misconceptions, stigma, socioeconomic vulnerability, practicing of self-medications, poor quality of services, and so on [3–6]. Studies in the context of communicable and noncommunicable diseases have highlighted that symptom burden, characterized by the subjective prevalence and frequency of symptoms, significantly affected health-seeking behavior [7–13]. However, there is a lack of quantitative estimates with regard to how the varying levels of symptom burden among people with presumptive TB contribute to their health care–seeking patterns in high–TB burden settings such as India or elsewhere.

In this background, the present study aimed to quantitatively assess the relationship between levels of symptom burden and health care seeking among people with presumptive TB by using a large-scale population-level survey conducted among persons with presumptive TB in a high–TB burden setting in South India.

## METHODS

### Study Setting

Data were derived from a population-level cross-sectional survey conducted between February 2021 and July 2022 in 32 districts of Tamil Nadu, a southern state of India. The state has a population of 70 million and >100 000 notified patients with TB during this period. The survey aimed to estimate the prevalence of microbiologically confirmed pulmonary TB and health-seeking behavior among the general population (≥15 years) in the state. All districts were selected to assess the prevalence estimates and health-seeking behavior for TB, ensuring representativeness of population from urban, rural, and diverse socioeconomic and geographic backgrounds. The presence of medical personnel, social workers, and community health workers ensured the conduct of a high-quality community-centric survey.

### Sampling

The survey was designed to recruit an expected 144 000 participants based on an average expected prevalence of pulmonary TB of 0.00119 with a relative precision of 0.4 for district-level variation. A design effect of 1.5% with 85% coverage was considered. Multistage cluster sampling was used to sample the participants across 180 clusters (cluster size, 800) via methods based on population proportionate to size. But this analysis was conducted among a subsample of this survey population who had reported symptoms presumptive of TB (supplementary appendix).

### Participant Screening Procedures

Study recruitment was conducted at the mobile field sites where the study team was stationed. The following criteria were used for screening purposes:

Inclusion criteria: age ≥15 years, resident in the selected village/urban census enumeration block for the previous month, available in the household during the period of fieldwork in the cluster
Exclusion criteria: institutional populations (schools, offices, prisons, defense establishments, hospitals, nursing homes, hostels, etc), nonconsenting hospitalized residents who were seriously sick and bedridden and unable to be radiographed and give sputum specimen

Eligible and consenting participants underwent TB symptom screening and history elicitation by a trained interviewer. Sociodemographic details, information on comorbidity, health care seeking for TB symptoms (present and past), health risk behaviors, and other contextual information were collected electronically. Presumptive participants were referred for diagnostic testing and/or clinical screening for TB (supplementary appendix).

### Study Variables

#### Outcome Measurement: Dependent Variable

Health care seeking for TB symptoms was considered the outcome measurement. It was defined as “participants who sought health care in either public sector or private sector health facilities or individual providers for specifically addressing their TB-related symptoms,” which included cough, expectoration or fever (for >2 weeks), blood in sputum (in past 6 months), chest pain, fatigue, loss of appetite, night sweats (>1 month), and weight loss (in the past 6–12 months).

Public sector facilities included primary health centers, community health centers, district hospitals, and medical college hospitals. Private sector facilities or providers such as allopathic and nonallopathic practitioners, chemists, private clinics, traditional healers, and trust hospitals were measured dichotomously as *yes* or *no*.

#### Covariates

To account for the factors that could affect the person's health care seeking, we adjusted for the following factors.

*Individual symptoms*. The presence of any 1 of the TB symptoms—cough, expectoration or fever (for >2 weeks), blood in sputum (in past 6 months), chest pain, fatigue, loss of appetite, night sweats (>1 month), weight loss (in past 6 to 12 months)—was measured dichotomously as *yes* or *no*. Persons with 1 or more symptoms were defined as presumptive TB.

*Symptom burden*. Symptom burden was measured at 4 threshold levels:

- Having ≥2 symptoms was defined as 1+ burden (coded 0) vs having only 1 symptom (coded 1).
- Having ≥3 symptoms was defined as 2+ burden (coded 0) vs having <3 symptoms (less symptom burden coded 1).
- Having ≥4 symptoms was defined as 3+ burden (coded 0) vs having <4 symptoms (less symptom burden coded 1).
- Having ≥5 symptoms was defined as 4+ burden (coded 0) vs having <5 symptoms (less symptom burden coded 1).

*Individual predisposing and enabling factors.* Age, gender, place of residency, and history of care seeking for TB were considered predisposing factors for health care seeking. Income status of persons with presumptive TB (categorized as quintiles) was defined as an enabling factor.

*Individual unhealthy factors*. Unhealthy factors associated with poor health care–seeking behavior included self-reported alcohol consumption and smoking and was categorized as *yes* or *no*.

*Population-level enabling factors*. Population-level access to health information and communication and access to health care facilities were computed as low and high level according to published secondary scores (S1Methods, supplementary appendix) [14, 15].

*Need-based factors*. The presence of diabetes and hypertension and a history of TB (yes or no) were added since they may have led to additional care needs.

### Statistical Analysis

The characteristics of the participants who were symptomatic were described by mean, SD, and 95% CI (via exact binomial formula) for continuous variables and by frequency, proportion, and 95% CI (via exact binomial formula) for categorical variables. The chi-square test was used to assess the association between all participant characteristics and symptom burden levels. We estimated multivariable logistic regression models to assess the association of symptom severity (1+ to 4+ severity level) with health care seeking after adjusting for individual TB symptoms and contextual factors. Adjusted odds ratios (aORs) with 95% CIs were calculated.

We performed Fairlie decomposition analysis, a most recognized method to quantify the contributions of intergroup differences in binary outcomes. In multivariate models, we estimated the contributions to differences in the association of a dependent variable of interest—health care seeking for TB symptoms—between 2 groups: presumptive TB cases with higher symptom burden (1+ to 4+) vs with less symptom burden. The Fairlie technique further decomposed the differences in the proportions between these types of presumptive TB based on the logit model. The explained difference was calculated as the sum of the differences in characteristics (ie, the values of the covariates) of the groups with high and low symptom burden, multiplied by the coefficients from the groups (with high symptom burden of 1+ to 4+) based on maximum likelihood estimation. A positive association explained the difference in decomposition means—that is, if the group with less symptom burden had the same characteristics as the group with higher symptom burden, then its health-seeking levels would be higher, and if negative, vice versa. The calculated probability was limited between 0 and 1. The estimated difference was further explained by observable covariates and unexplained differences [16–19]. As the analysis included only a subsample of surveyed participants with presumptive TB symptoms, cluster differences and variation were not considered. The analysis was performed in Stata/MP version 15.1 (Stata Corporation LLC) . Statistical significance was determined with a *P* value of .05 (2-sided; supplementary appendix).

### Patient Consent Statement

Written informed consent was obtained from eligible participants aged >18 years and parents/guardians of participants aged 15 to 18 years. Written assent was also obtained for participants aged 15 to 18 years. The study protocol was approved (017/NIRT-IEC/2021) by the Institutional Ethics Committee of ICMR–National Institute for Research in Tuberculosis, and the study protocol conforms to the 2017 national ethical guidelines for biomedical and health research involving human participants set by the Indian Council of Medical Research.

### Quality Assurance and Public Involvement

The survey protocol, design, and standard operating procedures were developed following the global guidelines of the World Health Organization. The recruitment and training of staff were done with standardized training modules (supplementary appendix). The study adhered to the STROBE checklist. The research question and objective of the proposed study were guided by past experiences of the investigators in conducting a TB prevalence survey in the community. During the development phase of the proposal, the study investigators were guided by community feedback on symptom screening mechanisms in the field. Extensive training and meetings were conducted in which community stakeholders participated. There were dedicated staff who undertook social mobilization and community engagement before the conduct of the survey activities in the field and enabled the community-centric nature of the study.

## RESULTS

### Presumptive TB and Health Care Seeking Among Participants

**Figure 1.**
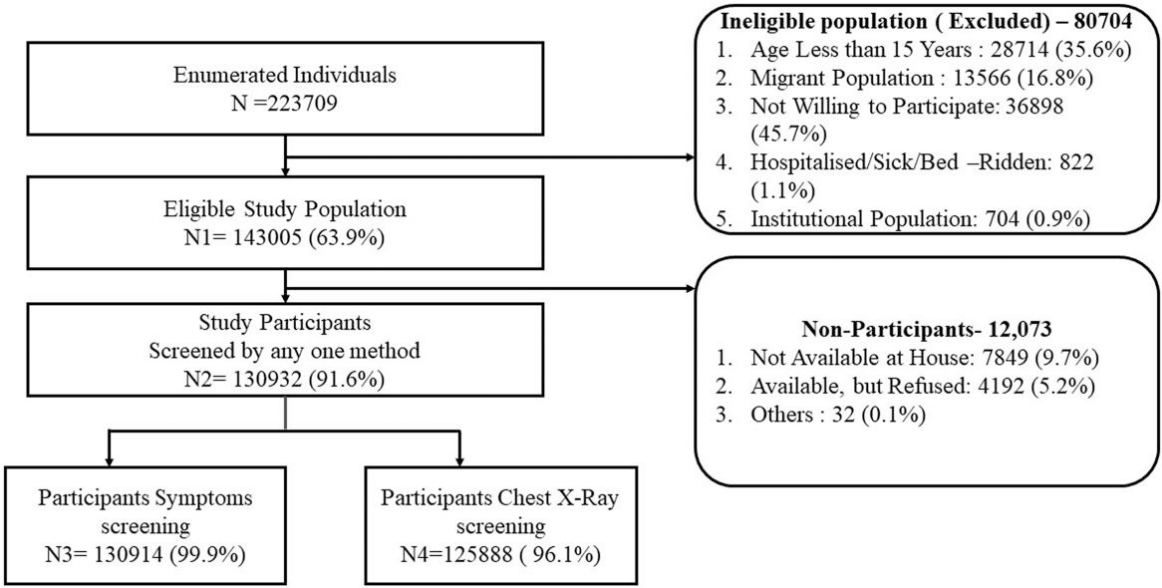
Flowchart depicting study enumeration and recruitment process. Participant screening and enrollment status.

Among the 223 709 people screened for the study, 143 005 (63.9%) were eligible. Of the remaining 80 704 who were ineligible, the reasons reported were as follows: age <15 years (n = 2 871 435.6%), migrant population that did not reside in the selected village/urban census enumeration block for the previous 1 month (n = 13566, 16.8%), not willing to participate (n = 36 898, 45.7%), hospitalized sick or bedridden (n = 822, 1.1%), and institutional population (n = 704, 0.9%). Among those who were eligible (n = 143 005), the majority (n = 130 932, 91.6%) consented and participated in the survey, and of the 12 073 who did not participate, the reasons reported were as follows: not available at home (n = 7849, 65.0%) and available but refused (n = 4192, 35%; Figure 1).

A total of 9540 people had at least 1 TB symptom, and among them, 6966 (73.02) reportedly did not seek care. After adjusting for those who sought care but were not able to continue (n = 104; due to reasons of unaffordability, delays in service delivery, etc), 2678 persons with presumptive TB (28.1%; 95% CI, 27.1%–28.9%) sought health care, whereas 6862 with presumptive TB (71.9%; 95% CI, 71.0%–72.8%) did not. Among those who accessed care for TB symptoms, 1614 (60.2%) have accessed care in public facilities, and 960 (35.5%) have accessed it in private facilities (supplementary appendix).

### Health Care Seeking by Sociodemography, TB Symptoms, Symptom Burden, and Contextual Factors

Results from the bivariate analysis showed that persons with presumptive TB who sought health care were more likely to be female; they had a higher income, a comorbidity, and greater access to health care facilities; and they had sought care for TB in the past (Table 1). The presence of cough, fever, weight loss, chest pain, appetite loss, expectoration, and fatigue was associated with increased health care seeking, but night sweats were not (*P* < .001). There was a significant difference in health care seeking between persons with increased symptom burden (across 1+ to 4+ levels) and those with less symptom burden (Table 2).

**Table 1.** Characteristics of Persons With Presumptive Tuberculosis by Their Health Care–Seeking Levels

	Health Care Seeking for Symptoms						
	No			Yes			
Characteristic	No.	%	95% CI	No.	%	95% CI	*P* Value^a^
Age, y							
15–24	574	8.4	7.7–9.0	220	8.2	7.2–9.3	
25–34	657	9.5	8.9–10.2	245	9.1	8.1–10.3	.81
35–44	977	14.2	13.4–15.0	384	14.3	13.1–15.7	
45–54	1257	18.3	17.4–19.2	476	17.7	16.3–19.2	
55–64	1488	21.6	20.7–22.6	627	23.4	21.8–25.0	
>64	1909	27.8	26.7–28.8	726	27.1	25.4–28.8	
Gender							
Male	3774	55.0	53.8–56.1	1408	52.6	50.7–54.4	
Female	3086	45.0	43.8–46.1	1269	47.4	45.5–49.2	.03
Residence							
Urban	3110	45.3	44.1–46.5	1283	47.9	46.0–49.8	.02
Rural	3752	54.7	53.4–55.8	1395	52.1	50.1–53.9	
Income							
Lower quintiles	3136	45.7	44.5–46.8	1125	42.0	40.1–43.8	.001
Higher quintiles	3726	54.3	53.1–55.4	1553	58.0	56.1–59.8	
Access to health information at district level							
Lower access	1737	25.3	24.2–26.3	707	26.4	24.7–28.1	.27
Higher access	5125	74.7	73.6–75.7	1971	73.6	71.8–75.2	
Access to health facilities at district level							
Lower access	5545	80.8	79.8–81.7	2036	76.0	74.3–77.6	.001
Higher access	1317	19.2	18.2–20.1	642	24.0	22.3–25.6	
Past care for tuberculosis							
No	6570	95.7	95.2–96.1	2377	88.8	87.5–89.9	.001
Yes	292	4.3	3.8–4.7	301	11.2	10.0–12.4	
Alcohol use							
No	5128	74.7	73.6–75.7	1980	73.9	72.2–75.5	.42
Yes	1734	25.3	24.2–26.3	698	26.1	24.4–27.7	
Smoking							
No	5364	78.2	77.1–79.1	2080	77.7	76.0–79.2	.59
Yes	1498	21.8	20.8–22.8	598	22.3	20.7–23.9	
Comorbidity^b^							
No	5557	81.0	80.0–81.8	2083	77.8	76.1–79.3	.001
Yes	1305	19.0	18.1–19.9	595	22.2	20.6–23.8	

^a^Chi-square test.

^b^Diabetes mellitus and hypertension.

**Table 2.** Health Care Seeking by Presence of Specific Tuberculosis Symptoms and Different Symptom Burden

	Health Care–Seeking Symptoms						
	No			Yes			
Characteristic	No.	%	95% CI	No.	%	95% CI	*P* Value^a^
Cough							
No	3125	45.5	44.3–46.7	1155	43.1	41.2–45.0	.03
Yes	3737	54.5	53.2–55.6	1523	56.9	54.98–58.7	
Fever							
No	6660	97.1	96.6–97.4	2566	95.8	94.9–96.5	.001
Yes	202	2.9	2.5–3.3	112	4.2	3.48–5	
Weight loss							
No	5424	79.0	78.0–79.9	2051	76.6	74.9–78.1	.001
Yes	1438	21.0	20.0–21.9	627	23.4	21.8–25.0	
Expectoration							
No	4215	61.4	60.2–62.5	1466	54.7	52.8–56.6	
Yes	2647	38.6	37.4–39.7	1212	45.3	43.3–47.1	.001
Fatigue							
No	5754	83.9	82.9–84.7	2126	79.4	77.8–80.8	
Yes	1108	16.1	15.2–17.0	552	20.6	19.1–22.1	.001
Appetite loss							
No	5397	78.7	77.6–79.6	1984	74.1	72.3–75.7	
Yes	1465	21.3	20.3–22.3	694	25.9	24.2–27.6	.01
Blood sputum							
No	6039	88.0	87.2–88.7	2349	87.7	86.4–88.9	.69
Yes	823	12.0	11.2–12.7	329	12.3	11.0–13.5	
Chest pain							
No	3986	58.1	56.9–59.2	1491	55.7	53.78–57.54	
Yes	2876	41.9	40.7–43.0	1187	44.3	42.45–46.21	.03
Night sweat							
No	5985	87.2	86.4–87.9	2387	89.1	87.8–90.2	
Yes	877	12.8	12.0–13.5	291	10.9	9.7–12.1	.01
Symptom burden^b^							
1+							
Yes	4220	61.5	60.3–62.6	1852	69.2	67.3–70.8	.0011
No	2642	38.5	37.3–39.6	826	30.8	29.1–32.6	
2+							
Yes	2133	31.1	29.9–32.1	1052	39.2	37.4–41.1	.001
No	4729	68.9	67.8–70.0	1626	60.7	58.8–62.5	
3+							
Yes	988	14.4	13.5–15.2	522	19.4	18.0–21.03	<.001
No	5874	85.6	84.7–86.4	2156	80.5	78.9–81.9	
4+							
Yes	498	7.3	6.6–7.8	249	9.3	8.2–10.4	.001
No	6364	92.7	92.1–93.3	2429	90.7	89.5–91.7	

^a^Chi-square test.

^b^Symptom burden 1+ is the presence of >1 tuberculosis symptom, whereas 2+, 3+, and 4+ denote having >2, >3, and >4 symptoms, respectively.

### Multivariable Logistic Regression

Persons with symptom burden 1+ were more likely to have sought care (aOR, 1.2; 95% CI, 1.0–1.3) as compared with persons with less symptom burden. Persons with symptom burden 2+ were more likely to have sought care (aOR, 1.3; 95% CI, 1.1–1.5) vs persons with less symptom burden. However, persons with a burden of 4+ were less likely to have sought health care (aOR, 0.7; 95% CI, .6–.9) than persons with less symptom burden (supplementary appendix).

### Net Difference in Health Care Seeking by Symptom Burden Levels

Findings show that health care seeking was 6.6 percentage points (95% CI, 4.8–8.4) higher for presumptive persons with symptom burden 1+ (model 1), 7.4 percentage points (95% CI, 5.5–9.3) higher for persons with symptom burden 2+ (model 2), 7.7 percentage points (95% CI, 5.2–10.2) higher for persons with symptom burden 3+ (model 3) and 5.7 percentage points (95% CI, 2.4–9.0) higher for persons with symptom burden 4+ as compared with persons with less symptom burden (Table 3).

**Table 3.** Fairlie Decomposition Results for Differences in Health Care–Seeking Levels Between Presumptive Persons With Higher and Lower TB Symptom Burden

	Model 1^a^			Model 2^b^			Model 3^c^			Model 4^d^		
	Percentage Points (95% CI)		Relative %^e^	Percentage Points (95% CI)		Relative %	Percentage Points (95% CI)		Relative %	Percentage Points (95% CI)		Relative %
Net health care–seeking difference	6.6 (4.8, 8.4)		100.0	7.4 (5.5, 9.3)		100.0	7.7 (5.2, 10.2)		100.0	5.7 (2.4, 9.0)		100.0
Component explained by measured covariates	3.0 (−2.3, 8.5)		45.9	2.1 (−4.9, 9.4)		29.0	6.7 (−2.1, 15.9)		87.2	10.6 (−4.4, 21.2)		184.2

	Explained differences of health care seeking											
Covariate	Absolute Difference (95% CI)	*P* > *z*	Relative % of total difference	Absolute Difference (95% CI)	*P* > *z*	Relative % of total difference	Absolute Difference (95% CI)	*P* > *z*	Relative % of total difference	Absolute Difference (95% CI)	*P* > *z*	Relative % of total difference
Age	−0.1 (−.3, .0)	.14	−1.9	−0.1 (−.3, .0)	.14	−2	−0.2 (−.4, .1)	.15	−2.3	−0.1 (−.3, .0)	.15	−2.3
Gender	−0.1 (−.1, .0)	.03	−1.1	0.0 (−.1, .0)	.19	−0.5	−0.1 (−.1, .0)	.08	−0.7	−0.2 (−.3, .1)	.00	−2.7
Residency	0.0 (.0, .0)	.51	−0.1	0.0 (.0, .0)	.14	0.3	0.0 (.0, .1)	.07	0.5	0.0 (.0, .0)	.73	0.1
Income status	0.1 (.0, .2)	.00	1.7	0.1 (.0, .2)	.00	1.4	0.1 (.0, .2)	.00	1.4	0.1 (.0, .2)	.01	2.2
Access to health information	0.0 (−.1, .1)	.91	−0.1	0.0 (−.2, .1)	.85	−0.2	0.0 (−.2, .1)	.82	−0.3	0.0 (−.3, .2)	.86	−0.4
Access to health facilities	0.1 (.1, .2)	.00	2	0.2 (.1, .3)	.00	2.6	0.1 (.1, .2)	.00	1.5	0.2 (.1, .3)	.00	3.6
Past care for TB	−0.2 (−.3, .2)	.00	−3.2	0.0 (.0, .0)	.42	0.1	0.6 (.5, .7)	.00	7.7	0.5 (.5, .6)	.00	9.5
Alcohol use	0.0 (−.1, .0)	.47	−0.3	0.0 (−.1, .0)	.36	−0.5	0.0 (−.1, .0)	.41	−0.4	0.0 (−.1, .0)	.60	−0.3
Smoking	0.0 (.0, .0)	.94	0	0	.79	−0.1	0.0 (−.1, .0)	.80	−0.1	0.0 (−.1, .1)	.83	−0.1
Comorbidity	−0.2 (−.2, .1)	.00	−2.3	−0.1 (−.2, .1)	.00	−1.7	−0.1 (−.2, .1)	.00	−1.7	−0.3 (−.4, .1)	.00	−4.6
Cough	0.4 (−.2, 1.0)	.19	6.1	0.3 (−.3, .9)	.33	3.9	0.7 (.1, 1.3)	.02	8.8	1.0 (.4, 1.5)	.00	16.4
Fever	0.3 (.1, .6)	.01	4.8	0.3 (.1, .6)	.01	4.2	0.4 (.1, .7)	.00	5.3	0.6 (.2, .9)	.00	9.7
Weight Loss	0.5 (.0, 1.0)	.07	6.9	0.5 (−.3, 1.2)	.20	6.4	1.1 (.2, 2.0)	.02	13.5	1.6 (.6, 2.7)	.01	28.3
Expectoration	1.3 (.6, 1.9)	.00	18.7	1.1 (.5, 1.8)	.00	15.3	2.0 (1.2, 2.8)	.00	25.2	2.7 (1.8, 3.6)	.00	46.3
Fatigue	0.9 (.1, 1.6)	.02	13.3	1.0 (−.1, 2.1)	.08	12.9	2.0 (.4, 3.5)	.01	25.2	3.1 (1.3, 4.9)	.00	53
Appetite loss	1.0 (.3, 1.6)	.00	14.5	1.1 (.1, 2.1)	.03	14.7	2.2 (.9, 3.5)	.00	27.5	3.1 (1.7, 4.5)	.00	52.6
Blood sputum	−0.1 (−.4, .2)	.60	−1.2	−0.3 (−.8, .2)	.32	−3.4	−0.1 (−.7, .6)	.81	−1	0.1 (−.6, .9)	.71	2.4
Chest pain	0.4 (−.3, 1.1)	.22	6.5	0.3 (−.7, 1.2)	.58	3.6	1.0 (.1, 2.0)	.04	13.2	1.5 (.5, 2.4)	.00	25.3
Night sweat	−1.2 (−1.7, .8)	.00	−18.6	−2.1 (−2.7, 1.4)	.00	−28	−2.8 (−3.8, 1.7)	.00	−36.2	−3.1 (−4.6, 1.6)	.00	−54.6

Dependent variables were health care seeking (yes = 0, no = 1). Symptom burden level was used as grouping variables in each model (yes = 1, no = 0). All covariates were used in the 4 models as independent variables.

Abbreviation: TB, tuberculosis.

^a^Model 1: presumptive TB with symptom burden 1+ level.

^b^Model 2: presumptive TB with symptom burden 2+ level.

^c^Model 3: presumptive TB with symptom burden 3+ level.

^d^Model 4: presumptive TB with symptom burden 4+ level.

^e^Relative percentage of total difference.

Model 1 shows that 45.9% of the difference in health-seeking behavior (ie, 3.06 of 6.68 percentage points) at symptom burden 1+ was explained by the sum of covariates, including individual symptoms and other contextual factors (ie, individual predisposing, risk, enabling, and need-based factors and population-level enabling factors). As symptom burden increased (through 2+, 3+, and 4+), 29.0%, 87.2%, and 184.2% of the difference in health care seeking was explained by the same set of factors (models 2, 3, and 4, respectively).

### Decomposition of the Difference in Health Care Seeking by Covariates

#### All Levels of Symptom Burden

**Figure 2.**
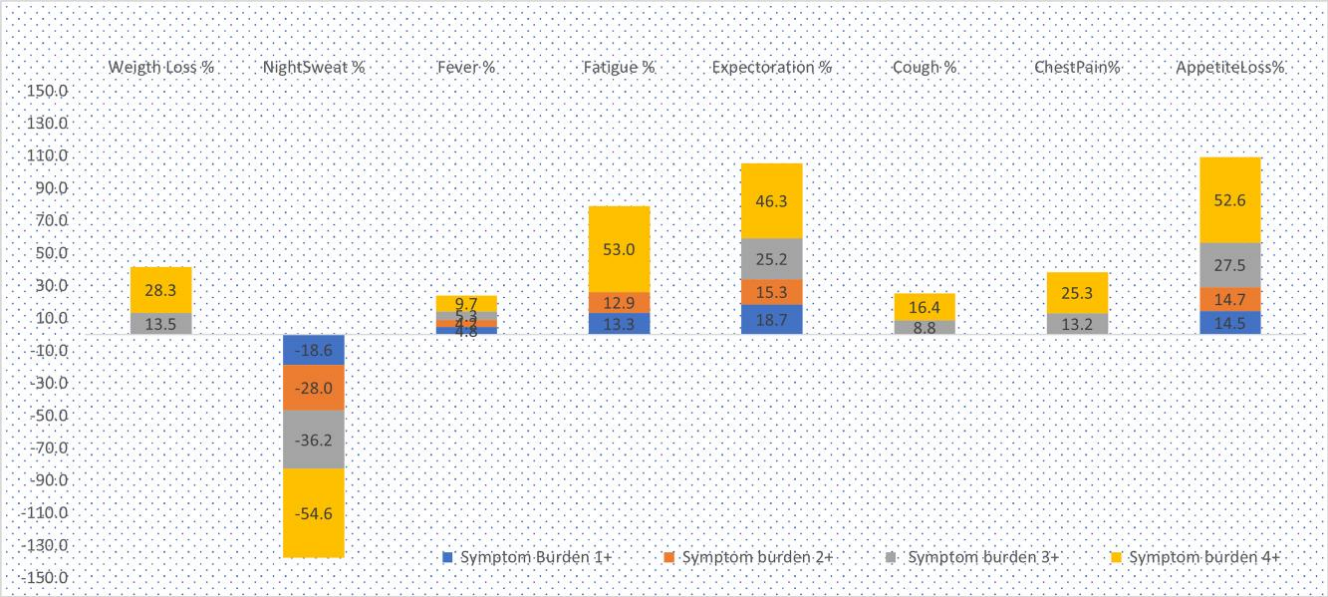
Contribution of individual tuberculosis symptoms in explaining health care seeking at different symptom burden levels.

At all levels of symptom burden (1+ to 4+), the presence of expectoration and loss of appetite had the largest positive contribution to health care–seeking differences (range, 1.0–3.1 percentage points; Table 3, Figure 2). The presence of fatigue had the largest contribution to health care seeking at symptom burden levels 1+, 3+, and 4+ (0.9–3.1 percentage points). Together the presence of these 3 symptoms highly contributed (46.5%–151.9%) to health care–seeking differences as compared with less symptom burden.

The presence of night sweats had the largest negative contribution to health care–seeking differences (percentage points [relative percentage], −1.2 [18.6%] to −3.1 [−54.6%]). The presence of comorbidity had a marginal negative contribution to health care–seeking differences (percentage points [relative percentage], −0.1 [−1.7%] to −0.2 [−4.6%]) as compared with less symptom burden. Higher income and increased access to health facilities marginally contributed (percentage points [relative percentage], 0.1 [1.4%] to 0.2 [3.6%]) to health care–seeking differences as compared with less symptom burden. At symptom burden levels 1+ to 3+, the presence of fever marginally contributed (percentage points [relative percentage], 0.3 [4.2%] to 0.4 [5.3%]) to health care–seeking differences, but at symptom burden 4+, fever moderately contributed (0.5 [9.7%]).

#### Symptom Burden at 3+ and 4+ Levels

At 3+ and 4+ symptom severity levels, the presence of cough (percentage points [relative percentage], 0.7 [8.8%] and 1.0 [16.4%], respectively), chest pain (1.0 [13.2%] and 1.5 [25.2%]), and weight loss (1.1 [13.5%] and 1.6 [28.3%]) moderately contributed to health care–seeking differences (Table 3, Figure 2). History of TB moderately contributed (percentage points [relative percentage], 0.5 [7.7%–9.5%]) to health care–seeking differences at symptom burden 3+ and 4+ levels but had a negative contribution (−0.2 [−3.2%]) at symptom burden 1+. Gender had a marginal negative contribution to health care–seeking differences at symptom burden levels 1+ and 4+ (percentage points [relative percentage], −0.7 [−1.1%] to 0.1 [−2.7%], respectively). Blood in sputum, unhealthy behaviors (alcoholism and smoking), and place of residence (urban and rural) did not have any significant contribution to health care seeking at any level of symptom burden.

## DISCUSSION

The study findings highlight symptom burden as a key factor that drives health care seeking among persons with presumptive TB. As symptom burden increased from 1+ to 3+, the difference in health care seeking increased. This positive association between symptom burden and health care seeking could be explained from an “illness perception” perspective, in which persons identify and associate a range of symptoms with their diseased condition [7–9, 20]. Illness perception is composed of one’s beliefs about the consequences of the disease and one’s personal ability to manage the disease condition. It is strongly influenced by symptom burden, severity, and clustering of symptoms in infectious and chronic disease conditions, which in turn mediate the health care–seeking behavior of the affected individuals [8–10, 21–24]. In addition, health care seeking had a marginal decrease at only symptom burden level 4+, which could be due to the mental and physical fatigue associated with multiple symptoms [24]. Despite this marginal decrease, we found that the contribution of measured covariates toward difference sin health care seeking was highest among those with 4+ symptom burden as compared with less severe symptom burden.

This study quantified that the most common symptoms of TB (cough, fever, blood in sputum, chest pain) moderately explained health care seeking. The findings of our study are consistent with past studies underscoring that cough and fever are usually perceived as minor and normalized health events and that health care is sought only as they turn chronic or distressing in association with other symptoms [5, 25–29]. Notably, a study conducted among a sample of 84 625 households in South India found that having a cough did not significantly increase the odds of care seeking [28]. Similarly, a large-scale prevalence study conducted in 17 states among tribal populations in India revealed that cough symptoms were not prompting care seeking [5]. The study also found that the presence of weight loss and chest pain did not contribute to health seeking at 1+ and 2+ levels of burden but made a moderate contribution at 3+ and 4+ burden levels.

Findings showed that symptoms such as expectoration and loss of appetite largely contributed to the difference in health-seeking behavior at all levels of symptom burden, while fatigue contributed to health care seeking at all levels of severity except 3+. Blood in sputum did not contribute to health care seeking at any level of symptom burden as noted earlier in Indian studies [5, 28]. Night sweats negatively contributed to care seeking at all levels of symptom burden, which deserves more exploration [30–32]. This larger contribution of a specific cluster of TB symptoms and health care seeking could be explained with the symptom appraisal theories used in chronic disease contexts, which underscore the role of symptom clusters in triggering care-seeking behaviors [33–36].

In this study, past TB treatment was not associated with health care seeking among presumptive persons when symptom burden was low. A lack of amnestic response and misperceptions about reinfections among patients with retreatment could explain this [37]. However, among persons with increased symptom burden, past TB care was only moderately associated with health care seeking. The presence of comorbidities decreased health care seeking marginally at all levels of symptom burden. Comorbid conditions could have normalized the illness perception concerning TB symptoms, which could explain its negative effects on care seeking. Gender differences had marginally decreased health seeking but not at all levels of symptom burden. Higher levels of access to health facilities and higher income status marginally increased health seeking, confirming past research [38].

Findings underscore the limitations of conventional IEC interventions (ie, information, education, and communication), which are delivered with an assumption to fulfill the community's lack of awareness about TB symptoms. Rather, IEC intervention for TB could be tailored to address the heterogeneity of symptom burden and its varied perception and interpretation among the population. TB awareness interventions also need to address cognitive-level barriers that lead to discounting or normalizing symptoms such as cough and fever among individuals who are affected. Evidence from COVID-19, HIV, and immunization interventions has shown that approaches such as targeted audience segmentation were useful in understanding population-level differences in risk perceptions, attitudes, and knowledge about symptoms and their patterns [39–41]. Developing tailored TB awareness and risk communication contents (eg, audiovisual, graphic, and interactive materials) for targeted engagement of populations and thereby improving their symptom interpretation could be useful [42–44].

The study draws its strength from its representative sample of a larger state of India, accounting for the differences in urban-rural geography, health infrastructure and services, socioeconomic status, and varied TB burden, which makes its findings generalizable. The large primary sample of persons with presumptive TB (n = 9540) identified in the community enabled us to arrive at population estimates of health-seeking behavior. The use of robust statistical techniques involving Fairlie decomposition with random subsampling helped us to arrive at the reliable estimates presented in this study. The study's limitations are that it was conducted during the COVID-19 pandemic, and the results should be interpreted cautiously. COVID-19 was noted to have improved infection prevention behavior in the community (eg, masking and cough hygiene) and thereby could have had an impact on the occurrence of respiratory infections and coughs during the period of our survey [45]. In addition, it has been widely noted that COVID-19 led to a reduction in health services and increased community hesitation for health care seeking for cough and fever symptoms. Both these factors could have confounded the estimates of health care seeking in this study. Also in the present study, symptom screening was carried out in an active survey mode in the community, which led to the early detection of symptoms; thus, the reported health care seeking for symptoms would be low. Studies conducted in low- and middle-income countries have shown that it takes patient more than a month to seek a diagnosis for TB symptoms [2, 46]. Our estimates of health care seeking for TB symptoms identified in active case finding mode corroborate the National TB Prevalence Survey data of India and earlier studies highlighting the generally low level of health seeking for TB symptoms in India [47, 48].

## CONCLUSION

Symptom burden of persons with presumptive TB was associated with their health care–seeking behaviors. The common and alarming symptoms of TB (cough, fever, and blood sputum) had a moderate contribution toward explaining differences in health care seeking, but a cluster of other symptoms (expectoration, fatigue, and loss of appetite) had a larger contribution in explaining the same. Thus, the subjective interpretation and appraisal of one's own symptom burden could be the plausible explanation for the difference in health care seeking among persons with presumptive TB. Developing and adopting a differential TB risk communication targeted at segments of the population with varied symptom burden and illness perceptions could improve health care seeking for TB.

## Supplementary Material

ofae412_Supplementary_Data

## Supplementary Data

Supplementary materials are available at *Open Forum Infectious Diseases* online. Consisting of data provided by the authors to benefit the reader, the posted materials are not copyedited and are the sole responsibility of the authors, so questions or comments should be addressed to the corresponding author.

## Notes

***Access to data and data analysis.*** P. G., K. N., S. S., K. T., A. F., and C. P. had full access to all the data in the study and take responsibility for the integrity of the data and the accuracy of the data analysis.

***Acknowledgments.*** We acknowledge all the participants of the survey, the local community leaders and administration for social mobilization, and all staff who conducted the survey; officials of the Health and Family Welfare Department–Tamil Nadu and the National Institute for Research in Tuberculosis–Chennai; and World Health Organization–National Tuberculosis Elimination Programme consultants, TB Prevalence Survey staff, and National Tuberculosis Elimination Programme staff who helped implement the survey. We also acknowledge the support given by various general health care staff, volunteers, and the communities that support the survey implementation. We also acknowledge the following members of the Tamil Nadu TB Prevalence Study Group: Havenesh Murugesan, Priya Rajendran, Makesh Kumar, Rajendran Krishnan, Paul Kumaran, J. Chitra, V. Rani, L. Venkatesan, P. Munivaradhan, D. Nithyakumar, V. Rameshbabu, P. K. Venkatramana, N. Premkumar, S. V. Joseph Rajkumar, T. Thangaraj, A. Devanathan, P. Balaji, T. K. Bharath, J. Udayakumar, Wilkingson Mathew, John Arockia Doss, A. Vasudevan, K. Anbarasan, M. Mahesh Kumar, P. Kumaravel, P. Chandrasekar, K. Vasudevan, G. Eswaran, R. Krishna Bahadur, J. Jeeva, E. Duraivel, R. Karunanidhi, and S. Kathiravan.

***Author contributions*.** Conceptualization: P. G., K. N., S. S., A. F., T. S. S., and C. P. Methodology: P. G., K. N., S. S., A. F., T. S. S., and C. P. Data collection and data curation: E. S., S. M., K. T. Data analysis: P. G., K. N., K. T. Writing–original draft preparation: P. G., K. N., C. P. Writing–review and editing: P. G., K. N., S. S., A. F., T. S. S., E. S., S. M., K. T., and C. P. Supervision and project administration: P. G., K. N., A. F. Resources and funding acquisition: T. S. S., A. F., P. G.

***Data sharing.*** A subset of the key anonymized individual participant data collected during the study, along with a data dictionary, is available upon request to the corresponding author, after approval of a proposal with a signed data access agreement.

***Disclaimer.*** The opinions expressed in this article are those of the authors and do not reflect the views of their employers or organizations. The funders had no role in the design and conduct of the study; collection, management, analysis, and interpretation of the data; preparation, review, or approval of the manuscript; and decision to submit the manuscript for publication.

***Financial support*.** This work was supported by the National Health Mission, Tamil Nadu, India.
