# Supplementary material for: Estimating and Explaining the Differences in Health Care Seeking by Symptom Burden Among Persons With Presumptive Tuberculosis: Findings From a Population-Based Tuberculosis Prevalence Survey in a High-Burden Setting in India
Source: Open Forum Infect Dis. 2024 Jul 19;11(8):ofae412. doi: 10.1093/ofid/ofae412 (PMC11310591; doi:10.1093/ofid/ofae412)
Supplement: ofae412_Supplementary_Data [file ofae412_supplementary_data.zip › Supplementary Appendix_Revision_Clean.docx]

**Supplementary Appendix -1**

This appendix has been provided by the authors to give readers additional information about their work.

Supplement to: : Giridharan P, Nagarajan K, Selvarauju S, Frederick A et al. Explaining the unknown relationship between symptom burden and health care seeking among persons with presumptive tuberculosis. Open Forum Infectious Diseases.

Supplementary Materials

Contents-------------------------------------------------------------------------------------2

**Supplementary methods**-----------------------------------------------------------------3

Study setting -------------------------------------------------------------------------------3

Sampling -----------------------------------------------------------------------------------3

Screening -----------------------------------------------------------------------------------3-4

Study variables ----------------------------------------------------------------------------5

Statistical Analysis Plan ------------------------------------------------------------------5-6

Data management cleaning and Quality Assurance -----------------------------------6

Addressing bias and confounding effects------------------------------------------------7

Supplementary Tables --------------------------------------------------------------------8-10

Supplementary References-----------------------------------------------------------------11

**Supplementary Methods**

**Study setting**

Tamil Nadu is a southern Indian state with a population of 70 million as per census 2011 The urban to rural population ration of the state was 48.4 to 51.6 % and had a population density of 555. The state has a total of 35 districts as on 2021 and is considered as one of developed state of the country. The state is ranked second in terms of health index among larger states in the country as on 2021. The state has a high burden of TB with the TB case notification rate as on 2021 was 100 per 100,000 population. The survey was conducted in close collaboration with the National TB Elimination program of Tamil Nadu state which covers all districts through its 461 TB units ( at subdistrict-level). [1-2]

**Sampling**

The calculated sample size was allocated to all 32 districts of the state (as per census 2011 of India) based on each district’s proportional contribution to the state population. In each units’ clusters were proportionately allocated (wards in urban and villages in the rural area). Selection of clusters in each unit was done as per ‘probability proportional to population size’ (PPS). Three CEB (Census Enumeration Block) as per census 2011 was used per cluster. Each CEB typically have 120–150 households which formed our clusters. Households in the sample area were updated and numbered. A random household was selected using Excel and two enumerators started enumeration from that household, in ascending and descending order till 400 eligible participants were identified by each enumerator. The enumeration included the details of all the family members in the household to ascertain the eligibility for participation. The study population included participants aged 15 years of age and above who resided in the selected cluster for at least one month. Hospitalized residents, institutional populations, participants who are refusing to give consent, seriously sick/bedridden were excluded. Eligible participants were given pre-printed Participant ID cards with QR codes which were linked to their IDs generated during the enumeration and referred to the survey site where the survey team were located. Reasons for non-participation among eligible participant was documented.

**Screening**

Participants having symptoms suggestive of TB (Cough > 2wks , Fever> 2wk , Blood in Sputum, Chest Pain> 1 month, Weight Loss, Loss of Appetite, Expectoration and Night Sweats), history of TB treatment (previous/current), and individuals having abnormal Chest X ray were eligible for sputum examination. ( Figure 1). Pregnant women were exempted from X-ray screening and were eligible for sputum examination only if they had symptoms. In addition to this, Chest X rays were also read by a panel consisting of 2 pulmonologists at the district level and if there were any discrepancy then a third umpire reading was done within 48 to 72 hrs to capture abnormality and thereby decide on the sputum eligibility. X ray abnormality reported by either the field medical officer or the panel was eligible for sputum examination. After the survey operations were over, the survey team shared the details of the participants diagnosed with TB with the local TB Program staff to start the participants on suitable treatment.

**Figure 1 Screening and testing strategy (algorithm) used in State TB prevalence survey, Tamil Nadu India, 2021-22**

All Eligible participants enrolled in TB Prevalence Survey

Interview for symptom screening, history elicitation using standard questionnaire

Chest X-ray PA view screening of all participants except pregnant women

Participants with :

- Symptoms suggestive of TB and/or
- Past history of TB and/or currently on TB treatment and/or
- Abnormal chest X-ray

Participants with :

- No symptoms suggestive of TB and
- No past history of TB and not currently on TB treatment and
- Normal chest X-ray

No sputum sample collected

Conditional 3^rd^ sputum if 1^st^ sputum is positive

First sputum specimen

Second sputum specimen

AFB-S microscopy in reference laboratory

CBNAAT for MTB in nearby CBNAAT lab

Liquid culture in reference laboratory

No TB

Third sputum specimen

Liquid culture in reference laboratory

CBNAAT for MTB in nearby lab

AFB-S microscopy in reference laboratory

No TB

TB +

TB +

No TB

TB +

No TB

TB +

No TB

TB +

No TB

TB +

***v) Population-level access to health information and communication***

A composite continuous measure of four factors (% of population with internet use, level of access to online governance, mobile subscription per 100 population and level of alternate sources of information) were categorised as quintiles in the first step. Further, the study districts which fell under the threshold of first quintile (<=55.8) were categorised as having low population access to health information and communication and districts with scores above 55.8 as having moderate or higher population access for the same.[3-4]

***vi) Population-level access to health care facilities in the study districts***

A composite continuous measure of three factors in the study districts (% of households with health insurance, percentage of households with no reported public health facility nearby, and number of public hospitals per 10000 population) were categorised as quintiles in the first step. Further, the study districts which fell under the threshold of the first quintile (<=0.128) were categorised as having higher access to healthcare facilities and districts with scores above 0.128 as having lower or moderate levels of access for the same.[3-4]

**Statistical Analysis Plan**

The estimates of TB symptom burden and health seeking were arrived after adjusting for cluster sampling. We used the Fairlie decomposition analysis method to estimate the net differences in the healthcare seeking of persons with presumptive TB due to the differences in their symptom burden levels. While the Fairlie decomposition method has been widely used to study race-related disparities, studies also have used the method to estimate disparities in treatment-related behaviour caused by symptom burden, which served as our reference. The average difference between groups with higher and lower symptom burden was expressed as:

$$\bar{Y}^{A}- \bar{Y}^{B}= \left[ \sum_{i=1}^{N^{A}} \frac{F\left( X_{i}^{A}\hat{\beta}^{A} \right)}{N^{A}}-\sum_{i=1}^{N^{B}} \frac{F\left( X_{i}^{B}\hat{\beta}^{A} \right)}{N^{B}} \right]+$$

$$\left[ \sum_{i=1}^{N^{B}} \frac{F\left( X_{i}^{B}\hat{\beta}^{A} \right)}{N^{B}}-\sum_{i=1}^{N^{F}} \frac{F\left( X_{i}^{B}\hat{\beta}^{B} \right)}{N^{B}} \right],$$

where $N^{j}$represents sample size for symptom burden group j (A=high symptom burden, B=less symptom burden), $\bar{Y}^{j}$is the mean probability of seeking health care for symptom burden j, $X_{i}^{j}$ represent the vector of independent variables with reference to case i in symptom burden j, $\hat{\beta}^{j}$ represents vector of coefficient estimates with inclusion of a constant. F represents the cumulative distribution function of the logistic distribution. Fairlie decomposition was based on pooled regression models which was run among symptom burden and their comparator i.e., symptom-less-burden persons whose sample size differed. Hence, we drew a random subsample of and match and rank the symptom burden groups based on their predicted probabilities. Since the results were sensitive to the subsample chosen; we bootstrapped for 1,000 different subsamples and final results calculated as a mean of values obtained from separate decompositions obtained from these subsamples. As the estimates were also sensitive to variable ordering in the decomposition equation, the order of variables was randomized across the simulations [5-6]. **Farilie** command in STATA software. We also assessed the potential correlation between the independent variables used in the model by running different ordinary logistic regression models (with dependent variable as health seeking for TB) and found that the variance inflation factors were less than 1.5 and ruled out multi collinearity.

**Data management cleaning and Quality Assurance**

Missing data was assessed for each variable of the study. For the overall survey population, a total of 18 participants didn’t respond to the questions of alcohol use, smoking, comorbidity and TB symptoms (missing value of less than 1%) and hence was included in the present analysis. Results of diagnostic X Ray was missing in 3.8 % of overall survey population but that variable was not included in the present analysis. But overall multiple imputation methods were used for addressing missing values while arriving at estimates. All the data collected in the field were synced with the local server, which was further transmitted to the central server located at NIRT, Chennai. All were entered in the online web application of the TB prevalence survey. Central Project Management Unit processed the data on daily basis and communicated with the field teams for ensuring quality assurance on real-time basis. The data management team at NIRT, Chennai cross-verified data that was collected in field (in android mobile devices), with the data synced with local van server at survey site and finally submitted to central server on completion of each cluster moving to next cluster. Once the field data entries were completed, the database was downloaded from the software. The data was cleaned and organized to meet the requirements of data analysis as suggested in the Tuberculosis prevalence surveys: a handbook by WHO [7]. Pilot surveys were carried out on sites. Based on the challenges identified during the pilot survey, the protocol, SOPs were modified. There was a survey steering committee which oversaw the progress of the study. Data entry was digitalized with a day-to-day data cleaning and feedback system to check data consistency was in place. The study confirms with STROBE checklist.

**Addressing bias and confounding effects**

Significant efforts were taken to mobilise the community for three days before the survey activity which resulted in significant participation from the community and to minimise over or under representation of the study general population. There was an 8% non -participation among the study eligible population, due to mostly reasons of non-availability and hence couldn’t be addressed. Overall, the refusal rate for the study was 5.2%, and considered to be minimal considering the larger study population of 143005. As the study collected self-reported data on health care seeking and risk behaviours among participants, there was a chance of bias in the reporting. The rigorous community mobilisation process enabled a positive and trusted environment in the community which enabled data collection in an effective way. Trained and experienced social workers and community workers who were involved in the interview process supported in collecting valid data. Our estimates of health seeking behaviour, alcohol and tobacco use corroborates with the range identified in earlier studies [8-11]. From the analysis point of view, the decomposition methods which we employed is considered effective in detecting inter-group differences in an outcome variable, while also calculating the contribution of other covariates to the outcome variable of interest. While calculating the relative contribution of symptom burden to the health seeking, the proportion of the explained differences and unexplained differences caused by symptom burden towards health care seeking was also calculated. This method was found advantageous than the conventional analytical approach( e.g. regression) in estimating the actual contribution of symptom burden towards health seeking.

Supplementary Table 1 Health care access by facility type for TB symptoms

| Type of facility | | n | % |
| --- | --- | --- | --- |
| Government facilities | PHC/Sub-Centre | 889 | 33.2 |
|  | CHC | 11 | 0.4 |
|  | District Hospital/Dis | 609 | 22.7 |
|  | Medical College | 83 | 3.1 |
|  | Other Government heal | 22 | 0.8 |
| Private facilities | MBBS practitioner | 691 | 25.8 |
|  | General Medicine practitioner | 143 | 5.3 |
|  | Chest specialist | 40 | 1.5 |
|  | Polyclinic | 20 | 0.7 |
|  | BAMS (Ayurveda) | 1 | 0.0 |
|  | BHMS (homeopathic) | 1 | 0.0 |
|  | Unani Doctor | 32 | 1.2 |
|  | Chemist | 23 | 0.9 |
|  | Traditional Healer | 2 | 0.1 |
|  | NGO/Trust | 7 | 0.3 |
|  | Missing | 104 | 3.9 |
|  | Total | 2678 | 100.0 |

**Supplementary Table 2: Multivariate regression of symptom burden level 1+ & 2+ with health care seeking among persons with presumptive Tuberculosis**

| Model 1 | | | | |  | Model 2 | | | | |
| --- | --- | --- | --- | --- | --- | --- | --- | --- | --- | --- |
|  | **AOR** | **[95% conf.** | | **P>z** |  |  | **AOR** | **[95% conf.** | | **P>z** |
| **Characteristics** |  |  |  |  |  |  |  |  |  |  |
| Symptom burden 1+ | 1.21 | 1.06 | 1.39 | 0.00 |  | Symptom burden 2+ | 1.31 | 0.00 | 1.11 | 0.00 |
| Cough | 1.06 | 0.96 | 1.18 | 0.23 |  | Cough | 1.05 | 0.39 | 0.94 | 0.38 |
| Fever | 1.42 | 1.11 | 1.81 | 0.01 |  | Fever | 1.40 | 0.01 | 1.10 | 0.00 |
| Weight Loss | 1.11 | 0.99 | 1.25 | 0.07 |  | Weight Loss | 1.08 | 0.21 | 0.96 | 0.20 |
| Expectoration | 1.22 | 1.10 | 1.35 | 0.00 |  | Expectoration | 1.21 | 0.00 | 1.09 | 0.00 |
| Fatigue | 1.17 | 1.03 | 1.33 | 0.02 |  | Fatigue | 1.13 | 0.09 | 0.98 | 0.08 |
| Appetite Loss | 1.19 | 1.06 | 1.33 | 0.00 |  | Appetite Loss | 1.14 | 0.03 | 1.01 | 0.03 |
| Blood Sputum | 0.96 | 0.84 | 1.11 | 0.63 |  | Blood Sputum | 0.93 | 0.34 | 0.80 | 0.33 |
| Chest Pain | 1.06 | 0.96 | 1.18 | 0.24 |  | Chest Pain | 1.03 | 0.62 | 0.92 | 0.62 |
| Night Sweat | 0.66 | 0.57 | 0.77 | 0.00 |  | Night Sweat | 0.64 | 0.00 | 0.55 | 0.00 |

Dependent variable is Health care seeking (coded 1 if yes 0 if No), Independent variable Symptom burden level (coded 1 if yes 0 if No). Each regression model was adjusted for covariates including i individual and population level factors which could pre -dispose , enable, necessitate or lower care seeking for TB

**Supplementary Table 3: Multivariate regression of symptom burden level 3+ & 4+ with health care seeking among persons with presumptive Tuberculosis**

| Model-3 | | | | |  |  | Model -4 | | | | |
| --- | --- | --- | --- | --- | --- | --- | --- | --- | --- | --- | --- |
|  | **AOR** | **[95% conf.** | | **P>z** |  |  |  | **AOR** | **[95% conf.** | | **P>z** |
|  |  |  |  |  |  |  |  |  |  |  |  |
| Symptom burden 3+ | 1.05 | 0.86 | 1.29 | 0.63 |  |  | Symptom burden 4+ | 0.77 | 0.60 | 0.98 | 0.04 |
| Cough | 1.12 | 1.01 | 1.24 | 0.03 |  |  | Cough | 1.16 | 1.05 | 1.28 | 0.00 |
| Fever | 1.48 | 1.16 | 1.89 | 0.00 |  |  | Fever | 1.54 | 1.21 | 1.97 | 0.00 |
| Weight Loss | 1.15 | 1.02 | 1.30 | 0.02 |  |  | Weight Loss | 1.20 | 1.07 | 1.36 | 0.00 |
| Expectoration | 1.28 | 1.15 | 1.42 | 0.00 |  |  | Expectoration | 1.34 | 1.21 | 1.48 | 0.00 |
| Fatigue | 1.19 | 1.04 | 1.37 | 0.01 |  |  | Fatigue | 1.26 | 1.10 | 1.45 | 0.00 |
| Appetite Loss | 1.22 | 1.08 | 1.38 | 0.00 |  |  | Appetite Loss | 1.28 | 1.14 | 1.44 | 0.00 |
| Blood Sputum | 0.98 | 0.85 | 1.14 | 0.84 |  |  | Blood Sputum | 1.03 | 0.89 | 1.19 | 0.69 |
| Chest Pain | 1.11 | 1.00 | 1.23 | 0.04 |  |  | Chest Pain | 1.15 | 1.05 | 1.27 | 0.00 |
| Night Sweat | 0.66 | 0.56 | 0.78 | 0.00 |  |  | Night Sweat | 0.71 | 0.60 | 0.83 | 0.00 |

Dependent variable is Health care seeking (coded 1 if yes 0 if No), Independent variable Symptom burden level (coded 1 if yes 0 if No). Each regression model was adjusted for covariates including i individual and population level factors which could pre -dispose , enable, necessitate or lower care seeking for TB

**References**

1)Directorate of Census Operation, Tamil Nadu.URL [https://census.tn.nic.in/PCA_data_highlights/executive_summary.pdf.](https://census.tn.nic.in/PCA_data_highlights/executive_summary.pdf.a) Accessed on 26.01.2023.

2)  Shewade HD, Frederick A, Kiruthika G, et al. The first differentiated TB care model from India: delays and predictors of losses in the care cascade. *Glob Health Sci Pract*. 2023;11(2):e2200505. <https://doi.org/10.9745/GHSP-D-22-00505>

3) Amit Kappor. Institute for Competitiveness : Social Progress Index. Districts and States of India.2022 Available at <https://eacpm.gov.in/wp-content/uploads/2022/12/Social_Progress_Index_States_and_Districts_of_India.pdf>.

4)Acharya R, Porwal A. A vulnerability index for the management of and response to the COVID-19 epidemic in India: an ecological study. Lancet Glob Health. **2020** 8(9):e1142-e1151.

5) Yang W, Li D, Gao J, Zhou X, Li F. Decomposing differences in depressive symptoms between older rural-to-urban migrant workers and their counterparts in mainland China. BMC Public Health. 2020;20(1):1442. Published 2020 Sep 23. doi:10.1186/s12889-020-09374-15)

6) Robert W. Fairlie. An Extension of the Blinder-Oaxaca Decomposition Technique to Logit and Probit Models. Discussion paper series IZA DP No. 1917. January 2006. URL . <https://docs.iza.org/dp1917.pdf>. Accessed on 26.01.2024

7)WHO. Tuberculosis prevalence survey: A handbook. ISBN 978 92 4 154816 8. 2007 URL. [https://iris.who.int/bitstream/handle/10665/44481/9789241548168_eng.pdf?sequence=1.](https://iris.who.int/bitstream/handle/10665/44481/9789241548168_eng.pdf?sequence=1.%20s)  Accessed on 26.01.2024

8) Rade, Kirakumar & Selvaraju, Sriram & Rao, Raghuram. (2022). India National TB Prevalence Survey report (2019-2021).

9) Suhadev M, Thomas BE, Raja Sakthivel M, et al. Alcohol use disorders (AUD) among tuberculosis patients: a study from Chennai, South India. PLoS One. 2011;6(5):e19485. doi:10.1371/journal.pone.0019485

10) Thomas BE, Thiruvengadam K, S R, et al. Smoking, alcohol use disorder and tuberculosis treatment outcomes: A dual co-morbidity burden that cannot be ignored [published correction appears in PLoS One. 2019 Nov 1;14(11):e0224914]. PLoS One. 2019;14(7):e0220507. Published 2019 Jul 31. doi:10.1371/journal.pone.0220507

11. Subbaraman R, Nathavitharana RR, Satyanarayana S, et al. The Tuberculosis Cascade of Care in India's Public Sector: A Systematic Review and Meta-analysis. PLoS Med. 2016;13(10):e1002149. Published 2016 Oct 25. doi:10.1371/journal.pmed.1002149
